# Untangling the diffusion signal using the phasor transform

**DOI:** 10.1002/nbm.4372

**Published:** 2020-07-23

**Authors:** Michael J. van Rijssel, Martijn Froeling, Astrid L.H.M.W. van Lier, Joost J.C. Verhoeff, Josien P.W. Pluim

**Affiliations:** ^1^ Center for Image Sciences, UMC Utrecht Utrecht the Netherlands; ^2^ Department of Biomedical Engineering Technische Universiteit Eindhoven Eindhoven the Netherlands

**Keywords:** diffusion fraction estimation, diffusion modeling, intravoxel incoherent motion, multi‐compartment diffusion modeling, phasor representation, tissue characterization

## Abstract

Separating the decay signal from diffusion‐weighted scans into two or more components can be challenging. The phasor technique is well established in the field of optical microscopy for visualization and separation of fluorescent dyes with different lifetimes. The use of the phasor technique for separation of diffusion‐weighted decay signals was recently proposed. In this study, we investigate the added value of this technique for fitting decay models and visualization of decay rates. Phasor visualization was performed in five glioblastoma patients. Using simulations, the influence of incorrect diffusivity values and of the number of b‐values on fitting a three‐component model with fixed diffusivities (dubbed “unmixing”) was investigated for both a phasor‐based fit and a linear least squares (LLS) fit. Phasor‐based intravoxel incoherent motion (IVIM) fitting was compared with nonlinear least squares (NLLS) and segmented fitting (SF) methods in terms of accuracy and precision. The distributions of the parameter estimates of simulated data were compared with those obtained in a healthy volunteer. In the phasor visualizations of two glioblastoma patients, a cluster of points was observed that was not seen in healthy volunteers. The identified cluster roughly corresponded to the enhanced edge region of the tumor of two glioblastoma patients visible on fluid‐attenuated inversion recovery (FLAIR) images. For fitting decay models the usefulness of the phasor transform is less pronounced, but the additional knowledge gained from the geometrical configuration of phasor space can aid fitting routines. This has led to slightly improved fitting results for the IVIM model: phasor‐based fitting yielded parameter maps with higher precision than the NLLS and SF methods for parameters f and D (interquartile range [IQR] for f: NLLS 27, SF 12, phasor 5.7%; IQR for D: NLLS 0.28, SF 0.18, phasor 0.10 μm^2^/s). For unmixing, LLS fitting slightly but consistently outperformed phasor‐based fitting in all of the tested scenarios.

## Graphical abstract

Using the phasor technique for separation of diffusion‐weighted decay signals was recently proposed. Here, we investigate its added benefit for fitting decay models and for visualization of decay rates. In the phasor visualizations of two glioblastoma patients, a cluster of points was observed that was not seen in healthy volunteers. For fitting decay models the usefulness of the phasor transform is less pronounced, but the additional knowledge gained from the geometrical configuration of phasor space can aid fitting routines, for example, in intravoxel incoherent motion.

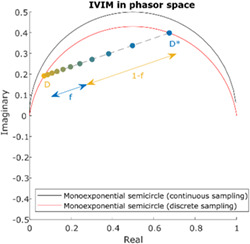

**Abbreviations used**
CSF: cerebrospinal fluid
CTV: clinical target volume
EPI: echo‐planar imaging
FLAIR: fluid‐attenuated inversion recovery
GTV: gross tumor volume
IVIM: intravoxel incoherent motion
LGE: late gadolinium enhancement
LLS: linear least squares
PDF: probability density function
SNR: signal‐to‐noise ratio

## 1 INTRODUCTION

The decay signal from diffusion‐weighted scans has been intensively studied and, consequently, the field has come up with a plethora of models to describe it, ranging from complex models based on physical descriptions of diffusing water molecules to relatively simple mathematical descriptions of the measured signal.^1^, ^2^ A common task is to separate the measured signal into components decaying at different speeds, such as fitting the intravoxel incoherent motion (IVIM) model or even more complex models that divide the diffusion signal into multiple water pools.^3^, ^4^ Recent works have shown that the use of IVIM model fitting is valuable for brain tumor characterization or when discerning brain tumor progression from pseudoprogression or radionecrosis.^5^, ^6^, ^7^, ^8^

Separating decay signals into two or more components can be challenging, especially in the presence of noise. Vergeldt et al applied so‐called phasor representation and subsequent signal separation to an in vivo diffusion dataset of the human brain.^9^ The phasor representation is generated by taking the Fourier transform of a signal and subsequently plotting the imaginary versus the real component at a single frequency, usually the lowest nonzero harmonic. This approach achieved promising results for fitting a three‐component model with fixed diffusivities (dubbed “unmixing”). The fraction maps generated with this approach may be valuable in a clinical setting or as a starting point for complex biophysical models.

**FIGURE 1:**
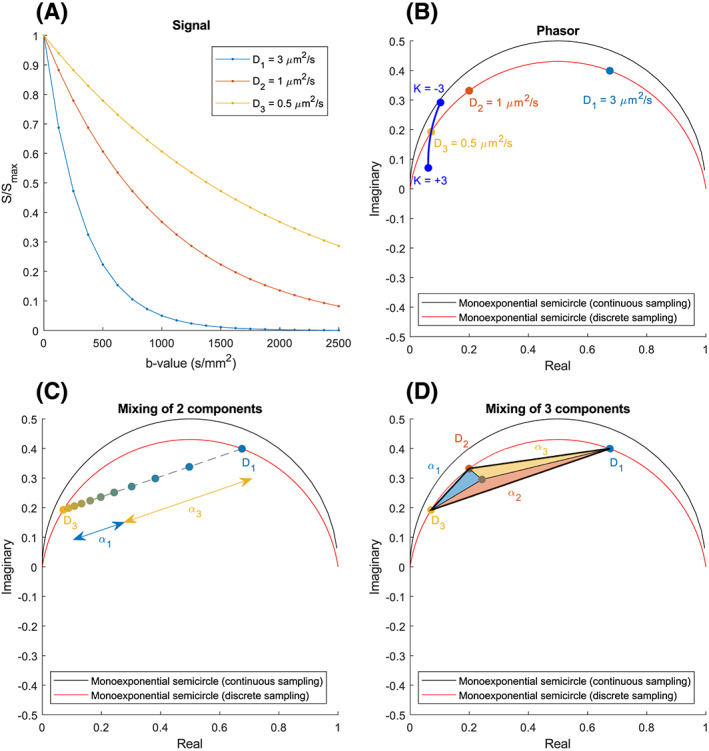
A, Simulated signal curves for three diffusivity values D, acquired using 21 equally spaced b‐values between 0 and 2500 s/mm^2^. B, Schematic phasor plot that indicates the position in phasor space of each of the curves in A using the same color scheme. The influence of diffusion kurtosis K was added using numerical simulations. C, Schematic phasor plot that shows the principle of two‐component mixing. The position of bi‐exponential decay curves in phasor space is on the line connecting the positions of the two pure components. The dots on the line indicate fraction increments of 10%. The colored arrows indicate phasor fraction α for a mixed signal with 60% component 1 and 40% component 2. D, Schematic phasor plot that shows the principle of three‐component mixing. The position of triple‐exponential decay curves in phasor space is inside the triangle connecting the positions of the three pure components. The colored triangles indicate phasor fractions α for a mixed signal with 40% component 1, 40% component 2 and 20% component 3

The phasor representation shows the position of an exponential decay curve in phasor space, which is determined by its lifetime, or a combination of lifetimes. Monoexponential decay curves are represented on a semicircle, with components possessing a short lifetime on the right and components with a long lifetime on the left of this semicircle. Inversely, from the position in phasor space, the lifetime of components can be estimated Figure 1.

Multiexponential decay curves, which can be regarded as mixtures of monoexponential decay curves, are represented in the area enclosed by the single‐exponential semicircle in phasor space. Bi‐exponential decay curves are found on the line segment connecting their two base components; the position on this line is determined by the weighting between the components. Triple‐exponential decay curves are found inside the triangle defined by their three base components, etc. These mixed signals can be unmixed (separated) from the sum signal into fractional contributions of up to a maximum of three components when fixing the lifetimes for those components across the dataset.^10^ A different approach with fixed lifetimes has been shown to have superior fit quality when compared with conventional fitting approaches.^11^

Although the phasor approach to unmixing of diffusion signals is simple and fast, it only allows for the unmixing of, at the most, three signals with known diffusivities. The same fraction maps can be obtained by a simple linear system inversion, but since the phasor transform effectively involves a noise reduction step by selecting the lowest nonzero frequency, the generated maps are expected to have a higher fit quality. Currently, the robustness of both phasor and linear inversion methods against noise and misestimation of the fixed diffusivities is unknown.

Transforming a dataset into phasor space has the additional benefit of producing a global visualization of all decay rates present in the data. By presenting a human brain diffusion dataset in phasor space using a 2D histogram, Vergeldt et al have extrapolated three major diffusivities: 3.1, 0.65 and <0.3 μm^2^/s (figure 5a in^9^). Although this separation into three diffusivities is a simplification of the full diffusivity spectrum present in the human brain,^3^ the underlying 2D histogram in phasor space represents a visualization of the full spectrum. This global visualization is potentially useful in detecting or diagnosing pathology in an efficient manner.

The phasor space may also be of use when fitting a more complex model, such as the IVIM model. The estimation of this model is often challenging since different combinations of parameters can yield model fits with comparable residuals.^12^ A popular approach to circumvent this instability is a two‐stage linear approach, often referred to as segmented fitting.^13^ This method, however, is prone to bias in parameter estimates.^14^ Since the IVIM model is bi‐exponential, the phasor transform might be of use in fitting this model, more explicitly by exploiting the property that bi‐exponential curves end up on the line connecting the two base components. This allows a reduction of the number of free parameters by two, which might yield more stable fitting results.

The present work investigates the usefulness of the phasor transform for both a global visualization of the decay rates in a diffusion dataset and fitting decay models. We test the hypothesis that using the phasor transform yields more stable fraction and parameter maps in both fixed‐diffusivity unmixing and IVIM model fitting. The influence of noise, component displacement and sampling on the accuracy of phasor unmixing is tested in a digital phantom. The performance of phasor unmixing is compared with that of linear unmixing as a benchmark. A phasor‐based IVIM fitting routine is developed and compared with both classical nonlinear fitting and linear segmented fitting in terms of accuracy and precision. The results of these simulations are compared with those obtained in a healthy volunteer. As a pilot study, global visualization is tested in five patients with biopsy‐proven glioblastoma.

## 2 THEORY

### 2.1 Phasor transform and decay rate estimation

In this section, we will provide a brief overview of the phasor representation. Both phasor transform and phasor unmixing have been presented in the field of optical microscopy for the separation of fluorescent dyes with different lifetimes.^15^, ^16^, ^17^ The existing phasor toolkit allows input signals that are equidistantly sampled over time. Since nonequidistant sampling across b‐values is very common in diffusion datasets, we will add an analytical description of phasor unmixing in case of nonequidistant sampling. This improves the general applicability of the phasor toolkit.

To maintain compatibility with existing literature on the phasor transform, this section describes single‐exponential signals with st=e−tτ, with time t and lifetime τ. In diffusion literature, the description $s\left(b\right)=e^{-\mathrm{bD}}$, with b‐value b and diffusivity D, is common. The translation from one framework to the other is easily achieved by substituting t with b and τ with 1/D in all of the equations below.

The phasor transform ℘ is defined by taking the Fourier transform of a signal s and dividing it by the sum of the same signal for normalization: $℘\left(s\left(t\right)\right)=\frac{F\left(s\left(t\right)\right)}{\int s\left(t\right)\mathrm{dt}}$. If the input is an exponential function of time t with lifetime τ, st=e−tτ, the phasor transform at frequency ω is the Lorentz function: $S\left(\omega \right)=\frac{1}{1+\mathrm{j\omega \tau}}$, with j the unit imaginary number. Plotting the real part of this function against the imaginary part for a fixed frequency ω for all possible lifetimes τ produces a semicircle with radius 0.5 and center (0.5, 0). Inversely, the decay rate of a signal can be estimated from its position in phasor space by inverting the Lorentz function: $\tau =\frac{j\left(S-1\right)}{\mathrm{S\omega}}$.

In the case of discrete equidistant sampling, the sampling rate will influence the appearance of the single‐exponential semicircle, deforming it into a semi‐ellipse. This phenomenon has been described by Fereidouni et al for retrospectively binned exponential decay in the context of optical microscopy.^18^ The authors provide an analytical description of this ellipse as a function of frequency, ω, and the number of measured samples or bins, U:

(1) $$S\left(\omega ,U\right)=\frac{\sum_{u=0}^{U-1}\left(e^{-\frac{u}{\tau }\;\frac{T}{U}}\left(1-e^{-\frac{1}{\tau }\;\frac{T}{U}}\right)\tau \right)e^{\mathrm{jn\omega}\left(u+\frac{1}{2}\right)\frac{T}{U}}}{\sum_{u=0}^{U-1}e^{-\frac{u}{\tau }\;\frac{T}{U}}\left(1-e^{-\frac{1}{\tau }\;\frac{T}{U}}\right)\tau }=\frac{e^{\frac{\text{jnTω}}{2U}}\left(e^{\frac{T}{\mathrm{U\tau}}}-1\right)}{e^{\frac{T}{\mathrm{U\tau}}}-e^{\frac{\text{jnTω}}{U}}}=\frac{\sinh\left(\frac{T}{2\mathrm{U\tau}}\right)}{\sinh\left(\frac{1-\text{jnωτ}}{\frac{2\mathrm{U\tau}}{T}}\right)},$$

with u the bin index, n the harmonic number and T the total sampling duration. The authors also describe a rotation of the deformed semicircle with decreasing U, which is due to their choice of the zero time point. For their case, each bin runs from t = (n‐1)T to t = nT, which is a useful choice since photons are sampled continuously and retrospectively binned. In diffusion MRI, diffusion decay is only sampled at the chosen b‐values and not in between. Consequently, we simply consider a discrete Fourier transform of a discretely sampled exponential decay divided by the discrete sum of the same signal, that is, the discrete phasor transform:

(2) $$S\left(\omega ,U\right)=\frac{\sum_{u=0}^{U-1}e^{-\frac{u}{\tau }\;\frac{T}{U}}\;e^{\text{jnωu}\frac{T}{U}}}{\sum_{u=0}^{U-1}e^{-\frac{u}{\tau }\;\frac{T}{U}}\;}=\frac{e^{\frac{T}{\mathrm{U\tau}}}-1}{e^{\frac{T}{\mathrm{U\tau}}}-e^{\frac{\text{jnTω}}{U}}}.$$

This describes a semi‐ellipse such as in the work of Fereidouni et al, but the rotation is no longer observed. An example of ellipse deformation due to discrete sampling can be seen in Figure 1B, which schematically shows the phasor plot of the single‐exponential discretely sampled signals shown in Figure 1A. Note that in this example and all the following analyses, we have chosen the lowest harmonic (*n* = 1), representing the lowest nonzero frequency after Fourier transformation. This is a sensible choice since the Fourier transform of a decaying signal has the highest magnitude there.^18^ This leads to the largest possible reference semicircle and the highest stability when unmixing. Additionally, Figure 1B shows the influence of diffusion kurtosis on the representation of a signal phasor space.^19^ Since signals that have nonzero kurtosis are not single exponentials, they are not represented on the semi‐ellipse. Instead, signals with positive kurtosis are represented in the area enclosed by the semi‐ellipse, while signals with negative kurtosis are represented above it.

When sampling is discrete and nonequidistant, as is often the case for b‐values in diffusion datasets, the single‐exponential semi‐ellipse will deform in an irregular way defined by the sampling pattern. This is not of concern when transforming data into phasor space or when determining the position of a decay rate on the circle since one can simply apply the phasor transform as defined. Although estimating the decay rate of a nonequidistantly sampled signal based on its position in phasor space has become nontrivial, this can be accounted for numerically.

### 2.2 Signal mixing in phasor space

Define a mixture m of single exponentials:

(3) mt=∑p=1Pape−tτp=∑p=1Papsτpt,

with τ_p_ the lifetime of the p‐th component contributing to the total signal with fraction a_p_. After applying the continuous phasor transform, the transformed signal can be described as a combination of the phasor transform of every single exponential:

(4) $$M\left(\omega \right)=\sum_{p=1}^{P}\alpha_{p}S\left(\tau_{p},\omega \right),$$

with M the phasor transform of the multiexponential signal m and α_p_ the weighting of the p‐th component in phasor space. α_p_ depends on the weights of all components in the time domain, a, and their associated decay rates, τ:

(5) $$\alpha_{p}=\frac{a_{p}\tau_{p}}{\sum_{p^{\prime }=1}^{P}a_{p\prime }\tau_{p\prime }}.$$

Graphically, this means that a mixed signal containing two components (ie, bi‐exponential decay) is located on a line connecting the locations of the two pure components in phasor space.^20^ The location of the signal on the line is determined by the weight α_p_. Analogously, mixtures of more components are located inside a polygon defined by the pure component vertices. An example of this process is shown in Figure 1C,D for two and three components, respectively. Note that this useful property of phasor space enables unmixing into the original signal fractions.

In the case of discrete sampling the mixing properties above still hold, but the weights of the components in phasor space now depend on the sampling pattern:

(6) $$\alpha_{p}=\frac{a_{p}\sum_{u=0}^{U-1}e^{-\frac{t_{u}}{\tau_{p}}}\;}{\sum_{p^{\prime }=1}^{P}a_{p\prime }\sum_{u=0}^{U-1}e^{-\frac{t_{u}}{\tau_{p\prime }}}\;},$$

with u the sample index, U the total number of samples, t_u_ the time at which the u‐th sample was measured, τ_p_ the lifetime of the p‐th component, a_p_ the signal fraction of the p‐th component and P the total number of single‐exponential components. This is a very general description that holds for all possible sampling strategies.

### 2.3 Phasor unmixing

Mixtures of signals containing up to three components can be unmixed using phasor unmixing.^10^ Treating the real and imaginary axes separately and enforcing that the sum of all components is 1, the unmixing of three components can be achieved by first solving:

(7) ReM=α1ReS1+α2ReS2+α3ReS3ImM=α1ImS1+α2ImS2+α3ImS3α1+α2+α3=1,

with S_p_ the phasor transform (ie, phasor coordinates) of signal s_p_ with corresponding phasor fraction α_p_, and M the phasor transform of the mixed signal. The solution of Equation 7 can be written in the form:

(8) α1=ImMReS2−ReS3+ImS2ReS3−ReM+ImS3ReM−ReS2ImS1ReS2−ReS3+ImS2ReS3−ReS1+ImS3ReS1−ReS2α2=ImMReS1−ReS3+ImS1ReS3−ReM+ImS3ReM−ReS1ImS1ReS2−ReS3+ImS2ReS3−ReS1+ImS3ReS1−ReS2α3=1−α1−α2,

which provides the phasor fraction, α_p_, for each component. To solve for signal fractions, a_p_, we have to invert Equation 6 for three components (*P* = 3):

(9) $$\left\{\begin{array}{c} a_{1}=\frac{\alpha_{1}E_{2}E_{3}}{E_{1}E_{2}+\alpha_{1}\left(E_{2}E_{3}-E_{1}E_{2}\right)+\alpha_{2}\left(E_{1}E_{3}-E_{1}E_{2}\right)}\\ a_{2}=\frac{\alpha_{2}E_{1}E_{3}}{E_{1}E_{2}+\alpha_{1}\left(E_{2}E_{3}-E_{1}E_{2}\right)+\alpha_{2}\left(E_{1}E_{3}-E_{1}E_{2}\right)}\\ a_{3}=1-a_{1}-a_{2} \end{array}\right),$$

with E_p_ defined as:

(10) $$E_{p}=\sum_{u=0}^{U-1}e^{-\frac{t_{u}}{\tau_{p}}}.$$

Note that although the sum of all components is set to be 1 in Equation 7, individual components are not restricted. Consequently, solutions for both a_p_ and α_p_ outside the range [0, 1] can be returned.

### 2.4 Phasor‐based IVIM fitting

The geometric properties of phasor space can be exploited to reduce the number of free parameters when fitting an IVIM model. The IVIM model assumes a sum of two single‐exponential signal components^4^:

(11) $$S\left(b\right)=S_{0}\left(\left(1-f\right)e^{-\mathrm{bD}}+fe^{-bD^{*}}\right),$$

with S the measured signal, b the b‐value, S_0_ the baseline MR signal, D the diffusion coefficient, D* the pseudodiffusion coefficient and f its associated fraction. In a classical nonlinear least‐squares fitting strategy, one has to estimate values for all four parameters of the model: S_0_, D, D* and f.

By exploiting the geometric properties of bi‐exponential signals in phasor space, the number of free parameters can be reduced to one, either D or D*. As outlined at the beginning of this section, estimating S_0_ is bypassed by transforming the data into phasor space since the phasor transform is normalized by definition. As is shown in Figure 1C, all bi‐exponential signals are located on a line connecting the locations of the two pure components in phasor space. Consequently, if one of the pure diffusivities (D or D*) is chosen as the free parameter and estimated using an iterative approach, the other diffusivity (D* or D) and the fraction (f) can be deduced using this property and a projection onto the single‐component semi‐ellipse. Assume, without loss of generality, that the free parameter is D*. The location of a single‐exponential decay with diffusivity D* in phasor space, L_D*_, is on the semi‐ellipse and can be calculated. The location of the measured signal, once transformed into phasor space, is L_S_. The other diffusivity (D) and the fraction (f) can be found by drawing a straight line **ℓ** in phasor space through L_S_ and L_D*_. The intersection of line **ℓ** with the semi‐ellipse on the other side of L_S_ gives us L_D_, the location of a single‐exponential decay with diffusivity D. Subsequently, D can be found by decay rate estimation, as detailed in section 3.1, and f can be found by unmixing, as detailed in section 3.3.

Note that section 3.3 describes unmixing for three components. As IVIM only has two components, α_3_ = 0, a_3_ = 0, and Equation 8 simplifies to:

(12) α1=ImS2ReM−ImMReS2ImS2ReS1−ImS1ReS2α2=1−α1.

Also, Equation 9 simplifies to:

(13) $$\left\{\begin{array}{c} a_{1}=\frac{\alpha_{1}E_{2}}{E_{1}+\alpha_{1}\left(E_{2}-E_{1}\right)}\\ a_{2}=1-a_{1} \end{array}\right).$$

## 3 METHODS

### 3.1 Phasor and unmixing

#### 3.1.1 Transforming data into phasor space

Phasor transformation of diffusion datasets was performed in MATLAB R2017b (MathWorks, Natick, MA, USA) on a desktop computer with a 3.50 GHz Intel Xeon E5–1620 v.3 central processing unit and 32 gigabytes of random access memory. First, data were sorted such that for every voxel location a sequence of data points with increasing diffusion weighting originated. Second, these sequences were transformed by applying a 1D inverse discrete Fourier transform using a fast Fourier transform algorithm (MATLAB's ifft function) along the diffusion‐encoding dimension. The resulting spectrum was divided by the sum signal for normalization. The phasor coordinates of each voxel were then determined by the real and imaginary components of the result at the lowest positive and nonzero frequency (ie, the lowest harmonic).

#### 3.1.2 Phasor unmixing

As outlined in the Theory section, the fractions of each pure component determine a voxel's coordinates in phasor space. Given the coordinates of these base components (component vertices) in phasor space, the relative signal fractions of up to three components can be estimated using phasor unmixing. A phasor unmixing routine was implemented in MATLAB that provides estimates for each fraction on a voxel‐wise basis, by taking each voxel's coordinates in phasor space and the coordinates of the component vertices and applying Equations 8‐10 (see also Figure 1D).

#### 3.1.3 Linear unmixing

Phasor unmixing was compared with the benchmark of linear unmixing. In linear unmixing, the fractions are calculated by directly solving Equation 3 for all fractions a_p_, while keeping all associated exponentials fixed. This is performed on the measured data in normal space. The linear system was solved using MATLAB's mldivide function.

### 3.2 IVIM model fitting

Three different algorithms to fit the IVIM model (Equation 11) parameters D, D* and f were compared: nonlinear fitting, segmented fitting and phasor‐based fitting. The nonlinear fitting was performed twice: once with and once without constraints on the fraction f.

#### 3.2.1 Nonlinear fitting

Nonlinear IVIM fitting was implemented using MATLAB's implementation of a trust‐region‐reflective least squares method. S_0_, D, D* and f were estimated using this solver and initialized at 0.1 μm^2^/s, 15 μm^2^/s and 0.5. D was constrained to be nonnegative, D* was constrained to be larger than or equal to 2.5 μm^2^/s, and f was constrained to be between 0 and 1. Additionally, D was constrained to be smaller than D*; the values of the parameters were switched in case the algorithm returned a result where D > D* and the value for f was adjusted accordingly.

#### 3.2.2 Segmented fitting

In segmented fitting, estimation was performed in two steps.^13^ In both steps, the fitted function was linearized by applying a log transform to the measured data. First, a single exponential was fitted to all data points from b‐values higher than 300 s/mm^2^. The perfusion component was considered to be negligible at b‐values higher than this threshold, so D was estimated directly from this fit. Next, the fraction f was estimated from the difference between the measured signal and the fit at b = 0, sidestepping the need to fit S_0_. Finally, the fit from the previous step was subtracted from the entire measured signal (using all data points) and D* was estimated by fitting a single exponential to the result.

#### 3.2.3 Phasor‐based fitting

Phasor‐based IVIM fitting was implemented using the projection technique described in section 3.4. D* was estimated iteratively on the measured data in normal space, using MATLAB's implementation of a trust‐region‐reflective least squares method.^21^, ^22^ D was deduced from phasor‐based symmetry and projection, as outlined in section 3.4. An estimate for f was obtained by phasor unmixing. D* was initialized at 15 μm^2^/s and constrained to be nonnegative. As in the nonlinear fitting method, D was constrained to be smaller than D*. A penalty term was added to the cost function to favor low fractions f (ie, monoexponential fits) for points close to the semicircle. Therefore, the cost function that was minimized is:

(14) $$\text{cost}=\left\{\begin{array}{cc} {\left(\hat{y}-y\right)}^{2}+{\left(\lambda \;f\;\left(1-\frac{L}{L_{0}}\right)\right)}^{2} & \text{for}\;L\leq L_{0}\\ {\left(\hat{y}-y\right)}^{2} & \text{otherwise} \end{array}\right),$$

with $\hat{y}$ the current model prediction, y the normalized measured signal, f the IVIM pseudo diffusion fraction, L the Euclidian distance to the semicircle, L_0_ the cutoff distance beyond which the extra penalty is zero, and the scaling parameter λ. The measured signal was arbitrarily normalized to the signal at b = 0 s/mm^2^; any b‐value can be chosen for normalization. L_0_ was set to 0.05 mm^2^/s, such that the extra penalty would only affect f‐values smaller than ~ 0.05. (A distance of 0.05 mm^2^/s from L_D*_ in phasor space roughly translates to f = 0.05 for the sampling scheme used.) λ was empirically set to 0.05, by manually balancing the error in f for true f‐values in the range 0‐0.25 by trial and error.

### 3.3 Digital phantom studies

#### 3.3.1 Unmixing analysis

A digital phantom was constructed, consisting of three diffusivity values loosely based on values generally found in the human brain^23^: 2.9, 0.85 and 0.18 μm^2^/s for D_1_, D_2_ and D_3_, respectively. The phantom contains increasing fractions for each component, consisting of 11 discrete steps increasing from 0 to 1 in steps of 0.1. Since the sum of all fractions must be 1, 66 unique combinations of fractions are simulated in this way. Each combination was simulated 10 000 times for different noise instances.

Diffusion‐weighted images were simulated with 21 b‐values equally spaced between 0 and 2500 s/mm^2^. Gaussian noise was added to both the real and imaginary parts of the complex signal resulting in four signal‐to‐noise ratio (SNR) levels of 30, 50, 100 and infinity (no noise added), where SNR is defined as average over standard deviation in the b = 0 s/mm^2^ image. Subsequently, the magnitude of the signal was taken, leading to a Rician noise distribution.^24^

The distribution of all simulated data in phasor space was visualized by creating two‐dimensional histograms. These histograms divided phasor space into square bins of 1/300 by 1/300 mm^4^/s^2^ and a color code was used to indicate the number of voxels present in each bin. To improve visibility, a logarithmic color code scale was used.

To investigate the dependency on noise, phasor unmixing was performed and the generated reconstructions were compared with the ground truth fractions. To investigate the influence of displacement of base components on the semicircle, the base components were systematically displaced, both in the presence of and in the absence of noise. Each of three base components was varied such that the diffusivity values used during unmixing varied from 0.75*D_true_ ‐ 1.25*D_true_ in steps of 0.05*D_true_. For every offset, the median error, as well as the 5%, 25%, 75% and 95% percentiles, were calculated.

The dependency on b‐value sampling was investigated by varying the number of b‐values from 3 to 99. This experiment was only performed with an SNR of 30 since this is a more realistic number in clinical practice.^25^

#### 3.3.2 IVIM analysis

Numerical simulations were conducted to generate signals with D set to 0.7 and D* set to 10 μm^2^/s; 101 fractions f in the range 0‐1 were simulated. Each fraction was repeated 5000 times with different Rician noise realizations at an SNR level of 30 at the b = 0 image. Fifteen b‐values were used, based on our in vivo acquisition (see section 4.4.2): 0, 10, 20, 30, 40, 60, 100, 150, 200, 250, 300, 400, 600, 800 and 1000 s/mm^2^.

The performances of the nonlinear, segmented and phasor‐based fitting algorithms described above were compared using the simulated data. For every simulated fraction, the median error as well as the 25% and 75% percentiles in the estimated parameters were calculated. Additionally, to facilitate comparison with our in vivo data, kernel density‐estimated probability density functions (PDFs) were calculated for parameters D and fat fraction levels closest to the levels that were found in the in vivo data: 0.07 for gray matter and 0.05 for white matter. These PDFs indicate the distribution of parameter estimates around the true value.

### 3.4 In vivo studies

#### 3.4.1 Scanning protocol and initial processing

All experiments were conducted after approval by the local ethical committee was obtained (NL53099.041.15 for the healthy volunteer, NL59820.041.17 for patient volunteers) and, before the exams, informed consent was obtained from all volunteers. A brain scan was obtained from one healthy male volunteer (aged 29 years) using a 3 T whole‐body MR system (Ingenia; Philips, Best, the Netherlands). The scan protocol contained a T_1_w ultrafast gradient echo as well as a diffusion scan with three orthogonal diffusion‐encoding directions and 21 b‐values ranging from 0 to 2500 s/mm^2^. The total acquisition time for the diffusion scan was ~ 10 minutes. A comparable diffusion‐weighted protocol with three orthogonal directions was added to the standard clinical protocol for five biopsy‐proven glioblastoma patients (aged 40‐83 years) in the context of imaging for radiotherapy planning, but with reduced feet‐head coverage of 30 2.5 mm slices around the tumor to limit the scan time to ~ 5 minutes. Details for each patient can be found in Table 1. Eight baseline scans with no diffusion weighting were acquired at regular intervals in between diffusion‐weighted scans to allow signal drift correction.^26^ Scan parameters per sequence are reported in Table 2. Additionally, from the standard clinical protocol, the fluid‐attenuated inversion recovery (FLAIR) and late gadolinium enhancement (LGE) images were obtained.

**TABLE 1:** Inclusion table with details of all included patients.

Patient	Figure	Age	Sex	Diagnosis	Remarks
1	7	83	M	Glioblastoma, gr IV, IDH wildtype	Biopsy only
2	S5	40	M	Glioblastoma, gr IV, IDH1 mutation	Biopsy only
3	S6	67	M	Molecular glioblastoma, gr IV, IDH wildtype	Biopsy only
4	S7	82	F	Glioblastoma, gr IV, IDH wildtype	Debulking
5	S8	43	F	Glioblastoma, gr IV, IDH wildtype	Debulking

Abbreviations: gr, World Health Organization grading of central nervous system tumors 2016; IDH, isocitrate dehydrogenase

**TABLE 2:** Overview of scan parameters per sequence. For every nonzero b‐value, the number of averages was 1 and three orthogonal directions were acquired

Sequence	TR (ms)	TE (ms)	Resolution (mm^3^)	FOV (mm^3^) (LR x AP x FH)	Bandwidth (Hz)	Fat suppression	b‐values (s/mm^2^)	Scan time (min)	Acceleration
*Healthy volunteer*									
3D T_1_w UGE	8	1.27	1.0 x 1.0 x 1.0	140 x 240 x 180	192	None	‐	4	*UGE factor*: 120 *SENSE*: 2 AP + 2 RL
DW SE‐EPI	8000	117	2.5 x 2.5 x 2.5	240 x 240 x 140	EPI:42, RO: 2067	SPIR	*	10	*EPI factor:* 39 *SENSE*: 2.5 AP
*Patient volunteers*									
DW SE‐EPI	4378	117	2.5 x 2.5 x 2.5	240 x 240 x 80	EPI:42, RO: 2067	SPIR	*	5	*EPI factor:* 39 *SENSE*: 2.5 AP

Abbreviations: AP, anterior‐posterior; DW, diffusion‐weighted; EPI, echo‐planar imaging; FH, feet‐head; FOV, field of view; LR, left‐right; RO, read‐out; SE, spin echo; SENSE, sensitivity encoding; SPIR, spectral presaturation with inversion recovery; 3D, three‐dimensional acquisition; T_1_w, T_1_ weighted; UGE, ultrafast gradient echo

* The list of scanned b‐values was: 0, 10, 20, 30, 40, 60, 100, 150, 200, 250, 300, 400, 600, 800, 1000, 1250, 1500, 1750, 2000, 2250 and 2500 s/mm^2^

Linear signal drift correction and correction for subject motion, eddy currents and echo‐planar imaging (EPI) distortion were applied to all diffusion‐weighted scans using available routines in ExploreDTI v.4.8.6.^27^ Motion and distortion correction was achieved by registering all diffusion scans to the T_1_w scan, downsampled to the resolution of the diffusion‐weighted scans. Registration involved affine registration in all directions for motion correction and b‐spline registration in the EPI direction for distortion correction using ExploreDTI's standard set of parameters. Probability masks for gray matter, white matter and cerebrospinal fluid (CSF), as well as a combined label image indicating the most probable tissue type per voxel, were obtained from the T_1_w image with SPM's Computational Anatomy Toolbox segmentation tool v.12.1 and subsequently downsampled to the resolution of the diffusion‐weighted scans.^28^ A brain mask was derived from the label image by selecting all voxels with a label corresponding to either CSF, white matter or gray matter. The brain mask was applied to all diffusion images before further analysis. Masks of each tissue type were created from the probability maps by selecting voxels with a probability higher than 0.99. Finally, geometric averaging was applied to all diffusion scans.

#### 3.4.2 Data analysis of the healthy volunteer

The nonlinear, segmented and phasor‐based IVIM fitting algorithms described in section 3.2 were applied to the acquired data of the healthy volunteer. Only b‐values of 1000 s/mm^2^ and below were selected for IVIM analysis. To compare the healthy in vivo data with our simulations, kernel density‐estimated PDFs were calculated for fitted parameters D and f inside the obtained gray and white matter masks. Parameter maps for all IVIM parameters were calculated for all fitting strategies. These maps were calculated using either all 15 b‐values of 1000 s/mm^2^ and below (0, 10, 20, 30, 40, 60, 100, 150, 200, 250, 300, 400, 600, 800 and 1000 s/mm^2^) or a subset of six b‐values (0, 30, 100, 250, 600 and 1000 s/mm^2^).

#### 3.4.3 Data analysis of the patient volunteers

Phasor analysis was performed on the data acquired from the glioblastoma patients, both on a reduced dataset containing all 15 b‐values of 1000 s/mm^2^ and below (c.f. healthy volunteer in section 2.4.2) and on the full dataset. On the phasor plot of the first patient created using the full range of acquired b‐values (0‐2500 s/mm^2^), a cluster of points was visible that was not seen on similar plots of healthy volunteers. This cluster was crudely delineated using a polygon and the corresponding voxels were obtained. For the four additional patients, the same analysis was performed using the polygon as defined in the phasor space of the first patient.

## 4 RESULTS

### 4.1 Unmixing

The phasor representation of the phantom data for the four simulated noise levels is shown in Figure S1 on a logarithmic color scale. Note that each of the 66 fraction combinations ended up in a unique location in phasor space. The spacing between increments in the fraction was nonlinear and the effect of noise differed per component.

Applying the phasor transform and unmixing a dataset containing 1 210 000 voxels and 21 b‐values took less than 0.5 seconds. Linear unmixing was slightly faster, occurring in just over 0.3 seconds.

**FIGURE 2:**
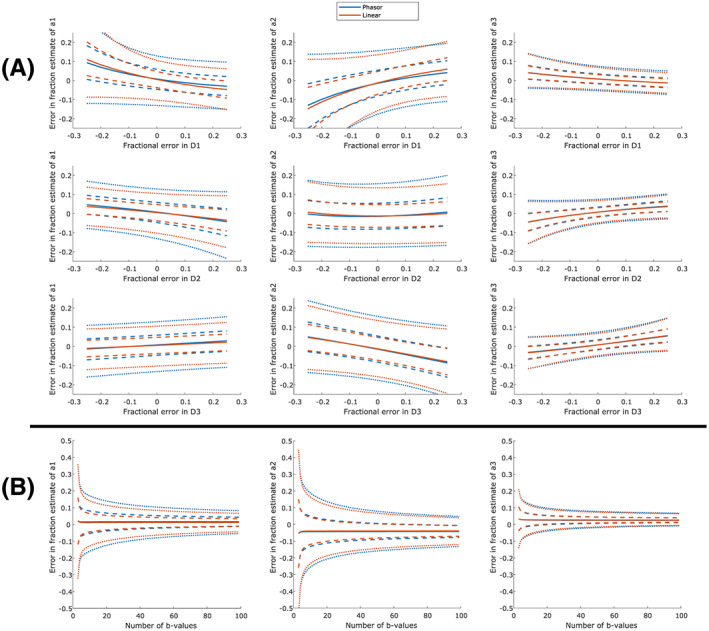
A, Influence of component vertex displacement on the fraction estimation by both phasor unmixing and linear unmixing in a simulated dataset with SNR 30. For every row, the influence of the displacement of one component's diffusivity (D1‐D3) on the fraction estimate of all components (C1‐C3) is investigated. The solid lines represent the median error, the dashed lines the 25% and 75% quantiles, and the dotted lines the 5% and 95% quantiles. B, Influence of the number of b‐values on the fraction estimation by both phasor unmixing and linear unmixing at an SNR level of 30

Figure 2A shows the accuracy and precision in the estimated signal fractions due to component misestimation for both phasor unmixing and linear unmixing. The noise‐induced bias at SNR level 30 for components 1, 2 and 3, respectively, was 0.016, −0.042 and 0.026 for phasor unmixing and 0.012, −0.039 and 0.027 for linear unmixing. Bias induced by component misestimation varied strongly depending on the displaced component. The maximum interquartile range due to a 10% error in diffusivity at infinite SNR was 0.045. The maximum interquartile range due to noise without displacement error was larger for SNR 50 and below (0.081 at SNR 50).

Figure 2B shows that linear unmixing slightly outperforms phasor unmixing across the entire range when the number of b‐values is varied. Both unmixing methods showed increased precision when the number of b‐values was increased, especially when the number of b‐values was low.

### 4.2 IVIM fitting

**FIGURE 3:**
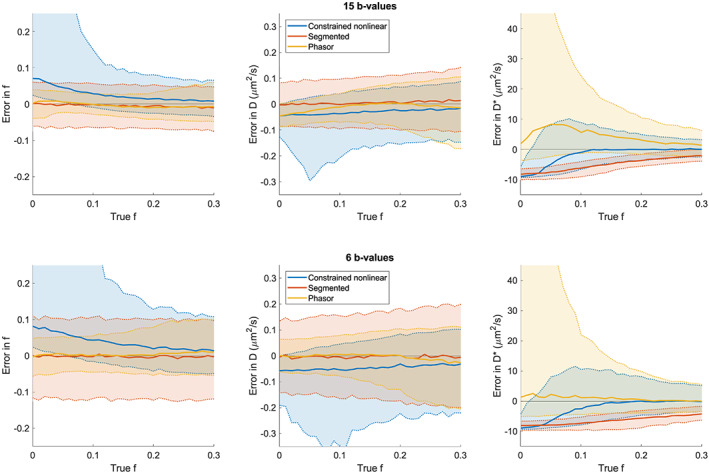
Comparison of IVIM fitting techniques on simulated data: constrained nonlinear least squares (blue), segmented linear least square (orange) and phasor‐based nonlinear least square (yellow). The SNR level was 30, and the b‐value sampling range was 0‐1000 s/mm^2^ for both experiments, but the number of b‐values was varied: 15 b‐values in the top panel and six b‐values in the bottom panel. The solid lines represent the median error and the dotted lines the 5% and 95% percentiles. Shading was added to facilitate a comparison of the spread in the errors

**FIGURE 4:**
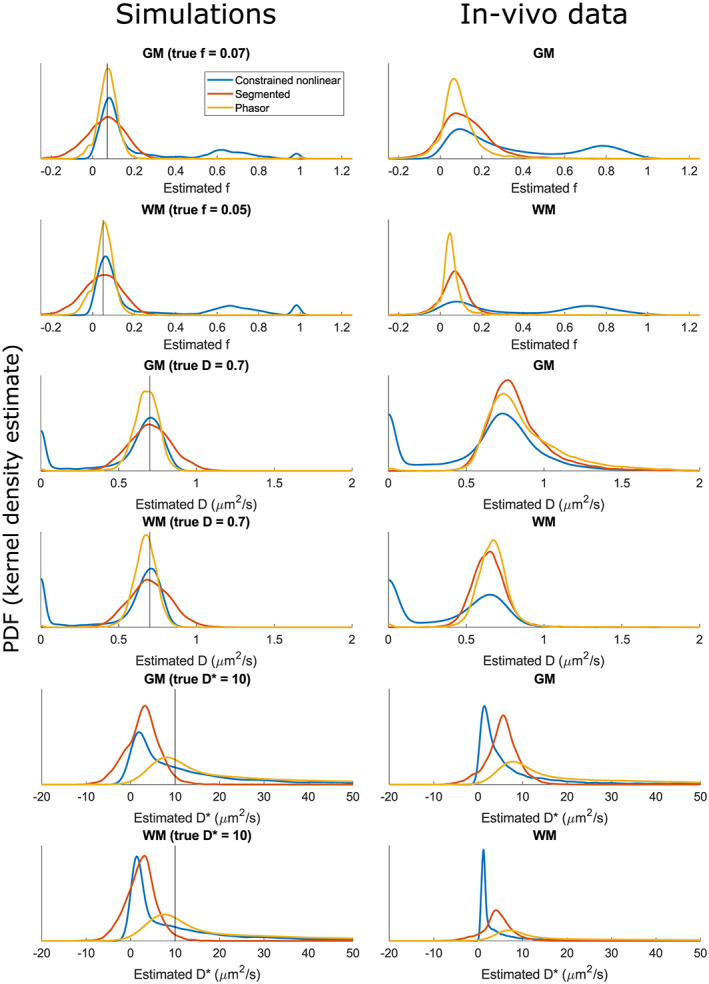
Probability density functions of estimated IVIM parameters f, D and D* for all tested fitting techniques in both simulated and in vivo measured white matter (WM) and gray matter (GM). In the simulations, the vertical lines indicate the true value of the estimated parameter. Results were obtained using all measured 15 b‐values

The runtimes of all tested fitting algorithms were compared: the phasor‐based fit used 2.38 x and the segmented fit used 0.0023 x the computation time of the NLLS IVIM fit. A comparison of the nonlinear, segmented and phasor‐based IVIM fitting methods on simulated data is presented in Figure 3 and the left panel of Figure 4. Figure 3 shows that for fractions f below 0.25 the phasor‐based method had the highest precision for estimates of f and D: the interquartile range was roughly halved compared with that of the segmented method, which was second best. Figure S2 shows the same data as Figure 3, but for the full range of f (0‐1). Accurately and precisely estimating D* at low fractions f proved difficult for all methods. The phasor method showed the best accuracy, but the worst precision. Table S1 reports mean and median error (accuracy) and interquartile range (precision) for all tested cases.

Figure 4 shows the PDFs of estimated values for f, D and D* for both simulated and measured white and gray matter. The simulation results for f and D showed that phasor‐based fitting had a higher precision than both nonlinear and segmented methods. In terms of accuracy, none of the tested methods was consistently the best, although both phasor and segmented outperformed the classical method by avoiding a local minimum with f ~ 0.7 and an unfeasibly low value for D. The accuracies of the phasor‐based and segmented methods were comparable. The simulation results for D* showed that although the phasor‐based method had the highest spread, it introduced the lowest amount of bias.

### 4.3 In vivo results

The in vivo results in Figure 4 show a similar distribution as the simulation results, although all distributions were slightly wider, most likely due to physiological variations in the imaged tissue included by the masks. The accuracy of the methods in vivo is hard to assess since no gold standard is available, but similar shifts in peak positions between different fitting strategies were observed for the in vivo results compared with the simulations. The phasor estimates had the lowest spread for all parameters, except for D in gray matter.

**FIGURE 5:**
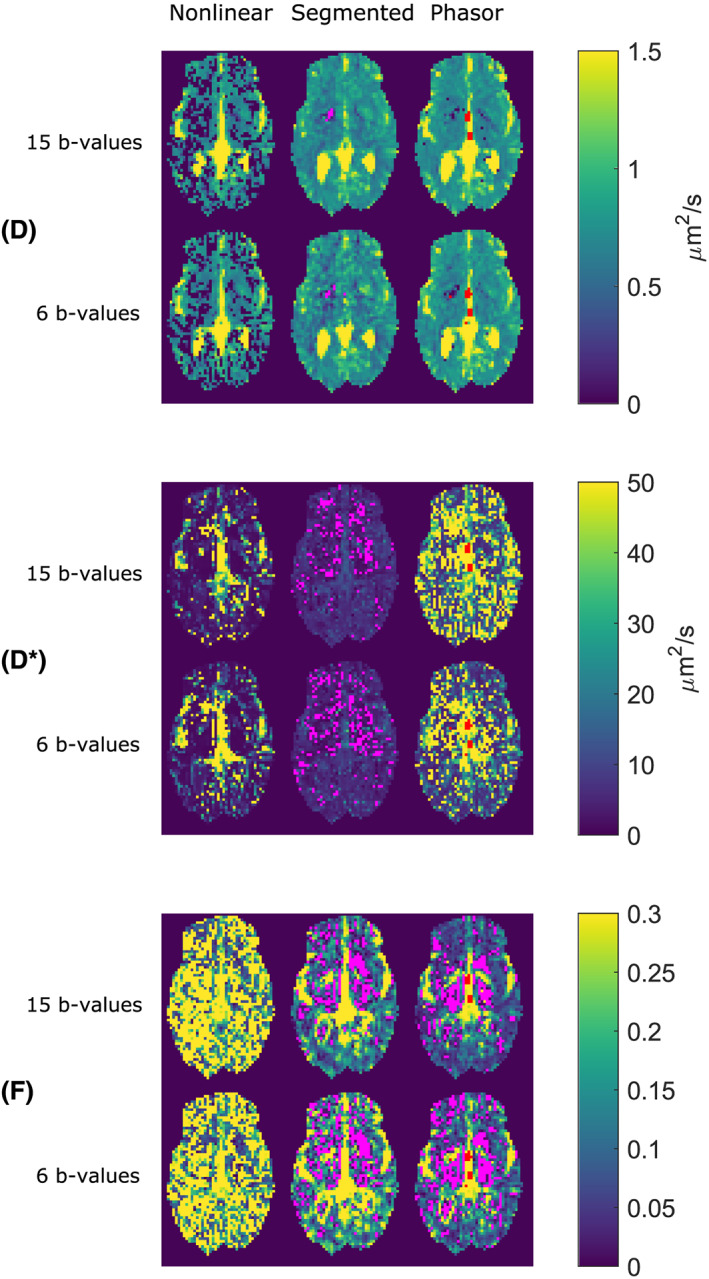
Comparison of IVIM fitting techniques on a diffusion dataset of a healthy volunteer with b‐values between 0 and 1000 s/mm^2^. For every parameter, there are two rows of maps: the top row shows maps calculated with all 15 b‐values, the bottom row shows maps calculated with a subset of six b‐values. Red voxels indicate locations where the algorithm failed to find a solution. Magenta voxels indicate locations where the algorithm returned physically implausible negative values

Figure 5 shows parameter maps for all IVIM parameters of the healthy volunteer, comparing those calculated with 15 b‐values with those calculated with six b‐values. Except for the (constrained) nonlinear method, all f maps showed an increase in the number of physically implausible negative values when decreasing the number of b‐values. These negative values were observed most often in central brain regions in white matter. The segmented method was the only method that returned negative values for D or D*. The D map calculated with the segmented method and only six b‐values contained a higher amount of variation than the same map calculated with the phasor method.

**FIGURE 6:**
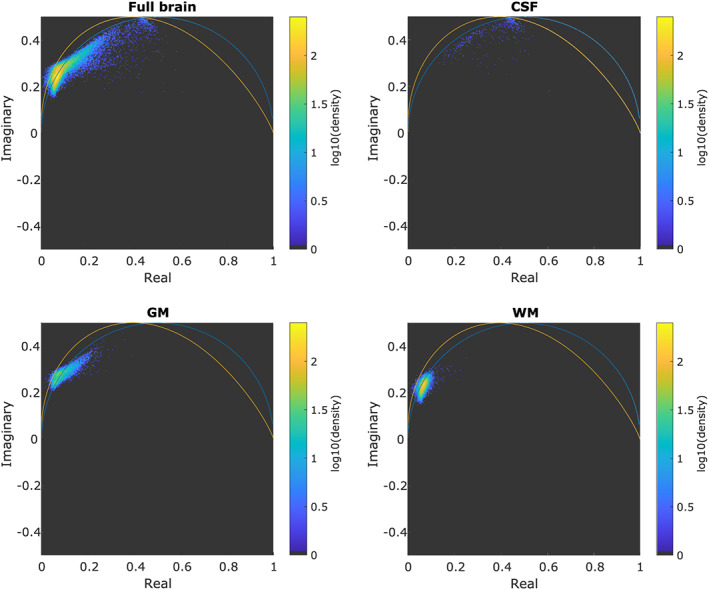
Phasor plots of a healthy volunteer for the b‐value range of 0‐2500 s/mm^2^. Different panels show full brain, cerebrospinal fluid (CSF), gray matter (GM) and white matter (WM) as indicated. The blue curve indicates the reference single‐component semicircle, the orange curve shows the single‐component curve for the sampling scheme used

**FIGURE 7:**
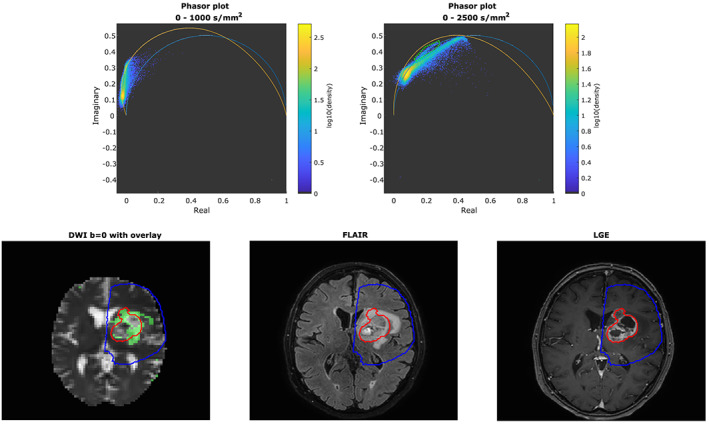
Phasor plots for 0‐1000 and 0‐2500 s/mm^2^ as well as b = 0 DWI, FLAIR and LGE images of the first glioblastoma patient. A cluster of points (encircled in green) is visible on the phasor plot of 0‐2500 s/mm^2^ that was neither seen in the healthy volunteer of Figure 5, nor in the literature.^9^ The corresponding voxels are indicated in the bottom‐left plot in green on top of the b = 0 DWI. The region seems to correspond roughly to a hyperintense region on the FLAIR image (bottom middle). For reference, the GTV and CTV are overlaid on the DWI, FLAIR and LGE images

Figure 6 shows the results obtained from the first glioblastoma patient. In the phasor space corresponding to the full dataset (0‐2500 s/mm^2^), a cluster of points, encircled in green, was observed that was not seen in healthy volunteers. For comparison, Figure 7 shows the phasor space of the healthy volunteer, both for the full brain and for segmented tissues. The pixels belonging to the discovered cluster are indicated in green in the bottom‐left panel and roughly corresponded to hyperintense regions on the FLAIR image of the same patient. For reference, the gross tumor volume (GTV) and clinical target volume (CTV) are overlaid on the observed cluster, FLAIR and LGE images. Additional data can be found in Figures S3‐S5, detailing the degree to which the same area can be found with conventional IVIM and kurtosis fits. Figures S6‐S9 show the same analysis as Figure 7, but for four additional patient volunteers.

## 5 DISCUSSION

This paper aimed to investigate the benefit of using the phasor transform for both global visualization of the decay rates present in a diffusion dataset and fitting decay models. Global visualization allowed fast identification of a cluster of voxels that roughly corresponded to the hyperintense region on the FLAIR images of two glioblastoma patients. Although the phasor transformation and unmixing yielded fast (sub‐second) estimation of signal component fractions, there was no clear benefit of phasor unmixing when compared with linear unmixing in terms of accuracy or precision. Both methods were found to behave similarly concerning the number of sampled b‐values, noise levels and component misestimation, with phasor‐based unmixing consistently performing slightly worse. IVIM model fitting seemed to benefit slightly from a phasor‐based approach: it featured a better precision than nonlinear fitting methods and the number of sampled b‐values influenced the outcome less than with segmented fitting.

### 5.1 Unmixing

Both component misestimation and the addition of Rician noise seem to cause a systematic bias in fixed‐diffusivity unmixing. Conversely, the number of b‐values hardly influences the accuracy. The effect of Rician noise is most apparent for high fractions of D_1_. Since D_1_ corresponds to the fastest decay (the highest diffusivity) in the simulation corresponding to free water, this signal is the fastest to reach the noise floor, where, due to the Rician noise distribution, the average signal is offset from zero. In phasor space, this leads to an offset from the expected position. This effect can be appreciated in the top right corners of Figures S1B‐D, where the group of pixels corresponding to a pure D_1_ signal deviates from its expected position on the semicircle. Note that this effect increases for decreasing SNR levels. The overall effect of Rician noise on the bias in fraction estimation is quite low; the absolute bias was always below 0.05. Additionally, as reported in Figure 2A, the bias induced by component misestimation was larger than the bias induced by Rician noise, even at small fractional errors and the lowest SNR level of 30.

The precision of fraction estimation was affected mainly by the SNR level and the number of b‐values. Component misestimation has a much smaller effect on the precision of the fraction estimate: a 10% relative error in diffusivity caused a smaller interquartile error range than adding Rician noise with an SNR of 50 or less. An increase in the number of b‐values causes an increase in estimation precision. As can be inferred from Figure 2B, this increase is most dramatic when the number of b‐values is low. Although the interquartile range decreases monotonically with an increasing number of b‐values, the improvements are very small from 10 b‐values and upwards.

Since phasor unmixing showed slightly worse accuracy and precision compared with linear unmixing for all tested cases, we can conclude that there is no intrinsic increase in fit quality when applying the phasor transform. The performance of phasor unmixing for multi‐component separation does not exceed that of linear unmixing. This leads us to conclude that the fit quality of fraction estimates obtained using phasor unmixing is entirely because the diffusivities are fixed.^11^ The transform itself, and its inherent noise reduction, do not contribute to an increase in fit quality.

### 5.2 IVIM fitting

Using the phasor transform can be beneficial since the transformed location of a signal provides additional knowledge due to the geometric properties of phasor space. The developed phasor‐based IVIM fitting technique serves as an example of this principle. Using the geometric properties of phasor space in a line‐projection strategy allowed a reduction of the number of free parameters to one. Additionally, proximity of a phasor‐transformed curve to the (deformed) semicircle is an indicator of monoexponential decay, which allowed a regularization based on the IVIM fraction and distance to the semicircle. This combination resulted in slightly more stable fitting results, where phasor‐based fitting consistently produced the highest precision for f and D in a limited set of simulations, as presented in Figures 4 and 5. No method consistently performed best in terms of accuracy. Either phasor‐based fitting or segmented fitting performed best depending on the simulated tissue, the evaluated parameter and the number of b‐values.

The PDFs of estimated IVIM parameters f and D are largely similar between the simulated and measured in vivo data when comparing the distributions in Figure 4. Since all variance in parameter estimation in the simulation can only stem from the added Rician noise, and given the observation that the distributions in vivo are only slightly wider, we conclude that noise is the dominant factor causing variance in IVIM parameter estimation in this in vivo brain experiment.

The parameter maps of the healthy volunteer in Figure 5 show that the most plausible D parameter maps are generated by the segmented and phasor‐based method; the maps generated by the nonlinear method contain many areas with implausibly low values. At those locations, the nonlinear fits converge to a local minimum with a very low D* value and a very high f. This tendency can also be observed in Figure 4, where the small peak for an estimated f around 0.7 corresponds with a peak for estimated D at very low values. Both phasor‐based and segmented methods avoid this local minimum. Based on our experiment with either 15 or six b‐values, the D maps generated by the phasor and nonlinear methods seem to be more robust against a low number of sampling points. The likely cause is that the phasor‐based and nonlinear fits use all data points to estimate D, while the segmented fit only includes data points above the threshold in this estimate. A lower number of required b‐values may be a valuable advantage in the clinic since acquiring fewer b‐values saves scan time.

All methods struggled to estimate D* accurately and precisely. Since the segmented method is linear, the segmented method is also the only method that is unconstrained for D* and frequently returns negative values, even if the value for D is positive. This is likely due to noise and the fact that D* is very hard to estimate. Additionally, the data points that are used for estimating D are more heavily influenced by the Rician nature of the noise since these are the data points corresponding to the highest b‐values and consequently the lowest amount of signal. An error in the estimate for D will propagate into the estimate for D*, which is estimated in the last step of the segmented method. This might be an explanation for the negative D* values found by the segmented method.

All methods, except the constrained nonlinear fit, returned negative values in the f parameter maps. These values were more frequent in central areas of the brain, mostly in white matter, where the fractions are expected to be lower. Furthermore, in these central areas, the SNR of the MR signal is lower due to a lower radiofrequency receive efficiency in these areas. Therefore, these slightly negative values most likely arise due to noise.

### 5.3 Visualization

The phasor plots of patients 1 and 2 (Figures 7 and S6) for b‐values up to 2500 s/mm^2^ contain a cluster of voxels that roughly corresponds to a hyperintense region on their FLAIR images. The cluster appears to correspond to the FLAIR‐enhanced edge region of the tumor and not to the core visible on LGE. On occasion, voxels are included that belong to CSF or the edge of the brain. More careful delineation of the cluster in phasor space might reduce these false positives. However, the locations of these voxels are known to be prone to movement and partial voluming, which negatively impacts analyses.

The cluster was not observed for patients 3‐5 (Figures S7‐S9). Patients 4 and 5 underwent surgery before their MRI, during which the LGE‐visible tumor was (partially) removed, which may explain the absence of the cluster. The reason for the absence of the cluster in patient 3 is not fully understood, but multiple explanations can be offered. First, patient 3 was diagnosed with a radiologically diffuse glioma, but with World Health Organization grade IV glioblastoma genotype. This type of tumor differs from typical glioblastomas, due to differences in its cellular structure.^29^ Second, patient 3 used corticosteroids (4 mg dexamethasone) at the time of the MRI, which may affect the diffusion properties of the tumor.^30^ Of note: patient 1 used the same medication at the time of the MRI and the effect of corticosteroids on diffusion properties may differ per tumor type.^31^ Future studies might include a larger cohort of patients to determine which clinical and biological factors contribute to the detectability of the cluster.

Since the cluster is not visible in the plot of b‐values up to 1000 s/mm^2^ and could not be separated using conventional IVIM parameters (Figure S4), this separation might indicate a change in diffusion kurtosis. The cluster plot of Figure S5 indeed suggests kurtosis is lower in the tumor cluster. This is in agreement with recent studies that have shown that gliomas have altered kurtosis parameters compared with normal‐appearing white matter and that measurements of mean kurtosis can distinguish low‐grade from high‐grade gliomas.^32^, ^33^ Including FLAIR hyperintensities in the CTV for radiotherapy planning is a matter of debate, since these regions can represent either residual tumor or edema.^34^ Current guidelines still recommend segmenting the GTV using a T_1_w contrast‐enhanced image and adding an isotropic 20 mm margin to define the CTV. A more refined definition of radiotherapy target volumes may lead to better local control and fewer side effects. Future studies might focus on whether phasor plots or kurtosis fits can aid in achieving a more direct estimation of the CTV volume by distinguishing between edema and residual tumor.

### 5.4 Study limitations

A limitation of this study is that the IVIM fit was tested only on simulated data, data of one healthy volunteer, and five glioblastoma patients. Therefore, the data distinguishing healthy subjects from patients using the observed cluster remains lacking in size. Consequently, the adoption of a “tumor cluster” in phasor space based on segmentation of the first patient is purely illustrative and a larger population consisting of both patients and healthy subjects would be needed to segment confidently in this way. A limited set of model parameters was tested and the simulated data contains only two components, while in in vivo data multiple components can be present. Most notably, free water was omitted from the simulations. The influence of three or more components on the IVIM fitting techniques was not investigated. However, when comparing the IVIM results of a healthy volunteer (right panel of Figure 4) to those of a two‐component simulation (left panel of Figure 4), there is no evidence to suggest that the phasor technique is differently affected than other techniques by the extra components present in the in vivo data. The influence of a wider range of brain pathologies on the calculated parameter maps might be of interest in a clinical setting. The aim of the present work, however, was to assess the added value of the phasor transformation in component separation techniques. The presented phasor IVIM fitting technique serves as an example of the possible benefit to be gained from this transformation. Future work may need to focus on improvements of phasor‐based fitting and rigorous evaluation of such a technique in a wide range of clinical cases, to validate the use of phasor‐based information in calculating parameter maps and to assess their clinical usefulness.

Since the observed benefit of phasor‐based IVIM model fitting is small, a conventional method with slightly different settings may still outperform the phasor method as presented. On the other hand, given the increase in performance observed by applying phasor‐based regularization to the problem, phasor information might be a valuable addition to new or existing methods. For example, it may be beneficial to estimate D* by a conventional least‐squares fit and use that estimate as a fixed D* in the phasor approach. With the experiments performed in this work, the benefit obtained by phasor‐based regularization cannot be separated from the benefit obtained by phasor‐based fitting. Future research may be conducted to separate those contributions and to identify the best way to incorporate phasor information in solving this or other fitting problems.

## 6 CONCLUSION

This paper aimed to evaluate the usefulness of the phasor transform for both visualization of decay rates and for fitting decay models. The phasor representation allows visualization and separation of tissues based on their diffusion properties and improves upon separation using conventional IVIM and kurtosis parameter maps. This has been demonstrated in a clinical setting in two glioblastoma patients. For fitting decay models the usefulness of the phasor transform is less pronounced, but the additional knowledge gained from the geometrical configuration of phasor space can aid fitting routines. A phasor‐based IVIM fitting routine yielded parameter maps with slightly higher precision than standard nonlinear methods. The usefulness of the phasor transform in unmixing signals when fixing the diffusivities is limited: a linear least‐squares routine slightly but consistently outperformed phasor‐based unmixing.

## Supporting information


**Table S1** Mean and median error (accuracy) and interquartile range (precision) for all IVIM parameters (f, D, and D*), all tested IVIM fitting strategies (nonlinear constrained, segmented, and phasor) and all simulated tissues (gray matter and white matter). Color codes indicate the best (green) and worst (red) results for easy interpretation. [Table S1 is submitted as a separate Excel‐file, TableS1.xlsx.]

**Figure S1** A: Phasor representation of simulated diffusion‐weighted images of a digital phantom consisting of three components with diffusivity values: D_1_ = 2.9, D_2_ = 0.85, D_3_ = 0.18 μm^2^/s. Each yellow dot corresponds to one of the 66 unique combinations of D_1_, D_2,_ and D_3_. B‐D: Phasor representation of the same phantom, but each of the 66 combinations was simulated with 10.000 unique noise realizations such that the SNR in the simulated b = 0 image was 100, 50, or 30 as indicated.

**Figure S2** [This figure shows the same as Figure 3 in the main paper, but for the full 0–1 range of the pseudodiffusion fraction f.] Comparison of IVIM fitting techniques on simulated data: constrained nonlinear least squares (blue), segmented linear least square (orange) and phasor‐based nonlinear least square (yellow). The SNR level was 30, and the b‐value sampling range was 0–1,000 s/mm^2^ for both experiments, but the number of b‐values was varied: 15 b‐values in the top panel and 6 b‐values in the bottom panel. The solid lines represent the median error and the dotted lines the 5% and 95% percentiles. Shading was added to facilitate a comparison of the spread in the errors.

**Figure S3** Results obtained from the diffusion dataset of the first glioblastoma patient volunteer with b‐values between 0 and 2,500 s/mm^2^.The top row shows the detected cluster of points in phasor space (left panel, encircled in red), the corresponding voxels are indicated in green on top of the b = 0 DWI (middle panel). The FLAIR image is shown for reference (right panel). The bottom row shows IVIM parameter maps of the same slice, calculated using the segmented fitting method. The region corresponding to the cluster can only be seen on the D parameter map as having a diffusivity higher than normal brain tissue, but lower than free water.

**Figure S4** Two‐dimensional histograms of the IVIM parameters fitted in the first glioblastoma patient using the segmented fitting method. The top row shows the entire brain on a blue color scale, the bottom row shows the same image of the entire brain with the data points corresponding to the detected cluster overlaid in red. The detected region also clusters in D vs f‐space but does not separate as clearly from the rest of the brain as it does in phasor space.

**Figure S5** Results obtained from the diffusion dataset of the first glioblastoma patient volunteer with b‐values between 0 and 2,500 s/mm^2^. The top row shows parameter maps resulting from an NLLS kurtosis fit of parameters K and D of the same slice as shown in Figure S3. The bottom left panel shows a two‐dimensional histogram of the fitted parameters for the entire brain on a blue color scale. The bottom right panel shows the same image of the entire brain with the data points corresponding to the detected cluster overlaid in red. The detected region also clusters in D vs K‐space but does not separate as clearly from the rest of the brain as it does in phasor space.

**Figure S6** [This figure shows the same as Figure 7 in the main paper, but for patient 2.] Phasor plots for 0–1,000 and 0–2,500 s/mm^2^ as well as b = 0 DWI, FLAIR and LGE images of glioblastoma patient 2. The polygon indicated in green in the top right panel is copied directly from patient 1.

**Figure S7** [This figure shows the same as Figure 7 in the main paper, but for patient 3.] Phasor plots for 0–1,000 and 0–2,500 s/mm^2^ as well as b = 0 DWI, FLAIR and LGE images of glioblastoma patient 3. The polygon indicated in green in the top right panel is copied directly from patient 1.

**Figure S8** [This figure shows the same as Figure 7 in the main paper, but for patient 4.] Phasor plots for 0–1,000 and 0–2,500 s/mm^2^ as well as b = 0 DWI, FLAIR and LGE images of glioblastoma patient 4. The polygon indicated in green in the top right panel is copied directly from patient 1.

**Figure S9** [This figure shows the same as Figure 7 in the main paper, but for patient 5.] Phasor plots for 0–1,000 and 0–2,500 s/mm^2^ as well as b = 0 DWI, FLAIR and LGE images of glioblastoma patient 5. The polygon indicated in green in the top right panel is copied directly from patient 1.
